# A Retrospective Analysis of Long-Term Prophylaxis with Berotralstat in Patients with Hereditary Angioedema and Acquired C1-Inhibitor Deficiency—Real-World Data

**DOI:** 10.1007/s12016-023-08972-2

**Published:** 2023-11-02

**Authors:** Felix Johnson, Anna Stenzl, Benedikt Hofauer, Helen Heppt, Eva-Vanessa Ebert, Barbara Wollenberg, Robin Lochbaum, Janina Hahn, Jens Greve, Susanne Trainotti

**Affiliations:** 1 University Hospital for Otorhinolaryngology, Head and Neck Surgery, Medical University of Innsbruck, Anichstraße 35, A-6020 Innsbruck, Austria; 2 Department of Otorhinolaryngology, Head and Neck Surgery, Klinikum rechts der Isar, TUM School of Medicine and Health, Department Clinical Medicine, Munich, Germany; 3 Department of Otorhinolaryngology, Head and Neck Surgery, Ulm University Medical Center, Ulm, Germany

**Keywords:** Acquired angioedema, Hereditary angioedema, HAE, C1-INH-deficiency, AAE-C1-INH, Berotralstat, Bradykinin, Prophylaxis, Long-term prophylaxis, LTP

## Abstract

Hereditary angioedema (HAE) and acquired C1-inhibitor deficiency (AAE-C1-INH) are orphan diseases. Berotralstat is a recently licensed long-term prophylaxis (LTP) and the first oral therapy for HAE patients. No approved therapies exist for AAE-C1-INH patients. This study is the first to report real-world clinical data of patients with AAE-C1-INH and HAE who received Berotralstat. All patients treated with Berotralstat were included in this retrospective, bi-centric study. Data was collected from patients’ attack calendars and the angioedema quality of life (AE-QoL) and angioedema control test (AECT) questionnaires before treatment, and at 3, 6, and 12 months after treatment and was then analyzed. Twelve patients were included, 3 patients with AAE-C1-INH, 7 patients with HAE type I, and 2 patients with HAE-nC1-INH. One patient (HAE I) quit treatment. Berotralstat was associated with fewer attacks in all groups. After 6 months of treatment, a median decrease of attacks per month was noted for HAE type I patients (3.3 to 1.5) and AAE-C1-INH patients (2.3 to 1.0). No aerodigestive attacks were noted for AAE-C1-INH patients. For HAE-nC1-INH patients, a mean decrease from 3.8 to 1.0 was noted (3 months). For HAE I patients, the total AE-QoL lowered a mean of 24.1 points after 6 months, for HAE-nC1-HAE patients 8.0 points, and for AAE-C1-INH patients 13.7 points. AECT scores increased for HAE I patients (mean: 7.1), HAE-nC1-INH patients (9.0), and AAE-C1-INH patients (4.2) after 6 months. Patients with HAE, HAE-nC1-INH, and AAE-C1-INH treated with Berotralstat showed reduced angioedema attacks and improved AE-QoL and AECT scores.

## Introduction

Acquired C1-inhibitor-deficiency (AAE-C1-INH) is a rare condition with a described prevalence of about 0.15:100,000 [1]. When studies are published, researchers are forced to report on small patient samples, leading to a lack of real-world data. Correspondingly, few studies have been performed investigating the efficacy of new medications towards treating these patients. Clinical trials testing efficacy of medications never include patients with AAE-C1-INH. As a result, no licensed therapy has been approved for AAE-C1-INH.

The disease is caused by an inappropriate consumption of the C1-INH associated with malignancy or through cleavage and inactivation of C1-INH by autoantibodies associated with autoimmune disorders. The pathophysiology of these disorders has not been fully defined, and patients with malignancy may also have autoantibodies to C1-INH [2, 3].

The resulting angioedema attacks cannot be clinically differentiated from those which occur in patients with hereditary angioedema (HAE), which has an estimated incidence of 1.5:100,000 [1]. Studies are regularly performed and treatment guidelines are available for C1-INH-HAE. However, no treatment guidelines are available and few studies are performed concerning AAE-C1-INH. Patients with AAE-C1-INH present with symptoms at an older age, typically older than 40 years, as compared to HAE patients [4]. Furthermore, AAE-C1-INH patients have a negative family history. A supporting diagnostic feature may be low levels of C1q which occur in about 70% of patients with AAE-C1-INH [2, 5].

Patients with HAE and AAE-C1-INH suffer from reoccurring angioedema attacks. These angioedema attacks may affect any cutaneous or mucocutaneous surfaces, including the extremities, face, genitalia, larynx, or gastrointestinal region. Attacks may be associated with hormonal fluctuations, physical trauma, infections, and stress, though they are often unpredictable. Swellings are painful and often lead to increased sick-leave. Additionally, laryngopharyngeal attacks may be deadly.

Berotralstat is currently the only licensed oral treatment for C1-INH-HAE patients aged 12 years or older approved by the FDA in December of 2020, and by the EMA in April of 2021. Berotralstat 150mg is taken once-a-day, and was shown to significantly reduce frequency of HAE attacks relative to placebo (respectively 1.65 vs. 2.35 attacks per month) over a 24-week period in the phase III APeX-2 trial [6]. The most common adverse effects include headaches as well as gastrointestinal complaints including abdominal pain and diarrhea. Published real-world evidence concerning Berotralstat is limited [7, 8]. A comparative meta-analysis juxtaposing prophylactic treatment using Lanadelumab given once every 2 or 4 weeks versus Berotralstat concluded that Lanadelumab has a superior effectiveness in reducing attacks within 28 days [9].

Treatment of the causative disease in patients with AAE-C1-INH is dependent on the underlying disease. In cases of low-grade hematological malignancy, the advantages of a therapy including potential side-effects of treatment are weighed against a watch-and-wait approach. Addressing the root cause of the disease is often – but not always - linked to symptom improvement [10–12]. In many cases, the underlying disease is an indolent lymphoma, which may not be deemed worthy of a chemotherapy. The adverse effects and toxicity caused by the chemotherapy are often worse than those symptoms caused by the indolent lymphoma itself. Additionally, a treatment-attempt via chemotherapy is also not a guarantee of success.

Frequently however, the underlying disorder cannot be effectively cured or even not found; in those cases, AAE-C1-INH may only be symptomatically treated. Currently, there are no licensed treatments by regulatory authorities for patients with AAE-C1-INH due to the small number of diagnosed patients and lack of clinical trials; therefore, treatment follows the therapy for HAE in an off-label fashion.

Approved therapies for patients with C1-INH-HAE may be stratified according to on-demand and prophylactic treatment. On-demand therapy includes the intravenously administered plasma derived C1-esterase inhibitor (Berinert) and (Cinryze), as well as the subcutaneously administered bradykinin B2 receptor antagonist (Icatibant) and the recombinant human-derived C1-esterase inhibitor (Conestat alfa). The subcutaneously administered kallikrein-inhibitor (Ecallantide) has been licensed from the FDA, though not from the EMA.

Prophylactic treatment options include intravenously administered plasma-derived C1-esterase inhibitor (Cinryze), subcutaneously administered plasma-derived C1-esterase inhibitor (Berinert), subcutaneously administered monoclonal antibody and kallikrein inhibitor (Lanadelumab), and orally administered kallikrein inhibitor (Berotralstat) [13].

The on-demand treatment with pdC1-INH has been shown to significantly reduce the duration of attacks in patients with AAE-C1-INH [14] and was shown to reduce frequency of attacks and improve quality of life in a case study [15]. When patients with AAE-C1-INH were treated for attacks on-demand with the bradykinin B2 receptor antagonist (Icatibant) or the kallikrein inhibitor (Ecallantide), an improved attack resolution was demonstrated [16, 17]. The kallikrein inhibitor Lanadelumab led to improved disease control and quality of life in patients with AAE-C1-INH [18].

Similar to patients with AAE-C1-INH, a lack of clinical trials and approved treatment options forces physicians to treat patients with HAE-nC1-INH according to their experience with patients with C1-INH-HAE. On-demand treatment with pdC1-INH substrate (Berinert) and bradykinin B2 receptor antagonist (Icatibant) has revealed reduced attack duration, time to symptom improvement, and attack intensity in smaller cohorts of 9–11 patients [19–21]. A smaller cohort of 3 patients described improved attack control after treatment with Lanadelumab [22]. In a case study, a reduction in attacks from 2 to 3 per month to zero was reported after 6 months of treatment with Berotralstat [23].

The primary goal of this study is to investigate the impact of treatment with Berotralstat 150mg on the frequency of angioedema attacks and quality of life as well as disease control. Patients with HAE-nC1-INH and AAE-C1-INH are rare subpopulations in which little or nothing is known about the effect of Berotralstat. Currently, no studies have described the use of Berotralstat in patients with AAE-C1-INH. This study documents the first described cases of treatment with Berotralstat in patients with acquired angioedema, and furthermore illustrates valuable real-world information. The secondary goal of this study is to report the impact of Berotralstat in patients with HAE-nC1-INH and AAE-C1-INH.

## Methods

The study protocol is in accordance with the Declaration of Helsinki. The institutional ethics board of the TUM School of Medicine and Health, Department of Clinical Medicine (Ethics committee file number: 2023–93-S-NP) and Ulm University (Ethics committee file number: 299/22) reviewed and approved the protocol.

In this bi-centric retrospective study, the patient medical database of the otolaryngology clinic of the Technical University of Munich School of Medicine, Klinikum rechts der Isar as well as the otolaryngology clinic of the University Hospital of Ulm—the two biggest angioedema centers in southern Germany—were searched for all patients who were treated with Berotralstat 150mg from April 1 2021, the first date that Berotralstat was approved by the EMA, to March 31 2023. All patients who had received Berotralstat were included in the study. Deidentified data points from patient files including age, sex, previous and current medical conditions, previously taken medications for angioedema, and records of angioedema attack calendars and AE-QoL and AECT surveys were entered into an Excel (V16.19) spreadsheet.

The AE-QoL (AE-QoL, Moxie GmbH, Berlin, Germany) survey is a validated tool used by physicians to help determine symptom base assessment of quality of life impairment in patients with recurring angioedema, independent of its underlying cause [24]. Higher scores are associated with a worse quality of life. A change of at least 6 points is considered to be the minimal clinically important difference (MCID). Statistical analysis was performed using Microsoft Excel for Mac (V16.19) and GraphPad Prism 5. A Wilcoxon matched-pairs signed rank test was used for statistical analysis in order to compare changes to AE-QoL and AECT before and after treatment.

## Results

### General Epidemiologic Data

A total of 3 patients (2 females) with AAE-C1-INH met the inclusion criteria and were switched to long-term prophylaxis (LTP) with Berotralstat 150mg once a day. Patients were aged 52 (male), 50 (female), and 87 (female). For the same observation period, a total of 9 HAE-patients (4 females) (median age 33 (male 31 years, female 40 years)) have been treated with Berotralstat among the two study centers. Among these patients 7 had HAE type I, whereas 2 had HAE-nC1-INH (see Table 1). Patients with HAE-nC1-INH received whole exome sequencing without a known mutation for HAE-nC1-INH being found. The diagnosis for patients P11 and P12 was based on clinical symptoms, lack of improvement to corticosteroids or antihistamines, and a quick and significant improvement in symptoms after receiving Icatibant or Berotralstat. P12 furthermore had a positive family history for angioedema.

**Table 1:** Demographic characteristics and available AECT and AE-QoL questionnaires for all patients (X available;—not available)

Nr.	Age	Sex	Angioedema type	AE-QoL questionnaires available for the months after start of Berotralstat					AECT questionnaires available for the months after start of Berotralstat				
				0	3	6	12	15–18	0	3	6	12	15–18
P1	52	m	AAE-C1-INH	X	X	X	-	X	X	X	X	-	X
P2	87	f	AAE-C1-INH	X	X	X	X	-	-	X	X	X	-
P3	50	f	AAE-C1-INH	X	X	X	-	-	X	X	X	-	-
P4	24	m	HAE type I	X	X	X	-	-	X	X	X	-	-
P5	36	m	HAE type I	X	X	X	X	-	X	X	X	X	-
P6	23	w	HAE type I	X	X	-	-	-	X	X	-	-	-
P7	47	w	HAE type I	X	-	X	-	X	X	-	X	-	X
P8	31	m	HAE type I	X	X	-	-	-	X	X	-	-	-
P9	12	m	HAE type I	X	X	-	X	-	X	X	-	X	-
P10	48	w	HAE type I	X	X	X	X	X	X	X	X	X	X
P11	33	m	HAE-nC1-INH	X	X	-	-	-	X	X	-	-	-
P12	33	w	HAE-nC1-INH	-	-	-	-	X	X	X	-	-	X

### Underlying Conditions as Potential Cause for AAE

For 2 AAE-C1-INH patients (P1 and P2), the diagnosis of MGUS (IgM κ and IgG λ) was made shortly after symptoms’ onset, while the male patient (P1) developed an indolent B-cell-lymphoma 3 years after the first symptoms of AAE-C1-INH began. Patient P3 undergoes yearly hematooncological and gynecologic as well as gastroenterologic checkup with no sign of causative disease.

### Previous Treatment Before LTP with Berotralstat for the Total Number of Patients (n = 12)

The initial on-demand-therapy (ODT) was Icatibant SC for the 3 AAE-C1-INH-patients. The severely affected male patient with MGUS (P1) had a period of LTP with pd-C1-INH-concentrate SC twice a week with no notable benefit. After the diagnosis of indolent B-cell lymphoma in July 2021, there was still no indication for a therapy according to the consulting hematologist.

The 9 HAE patients received Icatibant SC or pd-C1-INH-concentrate IV as ODT. Two patients with HAE type I additionally received a LTP with pd-C1-INH-concentrate IV once a week (P4) or twice a week (P9), while the 2 patients with HAE-nC1-INH (P11 and P12) had received LTP with Lanadelumab 300mg SC with loss of efficacy.

### Changes in Attack Rate (Before and After LTP with Berotralstat)

**Fig. 1:**
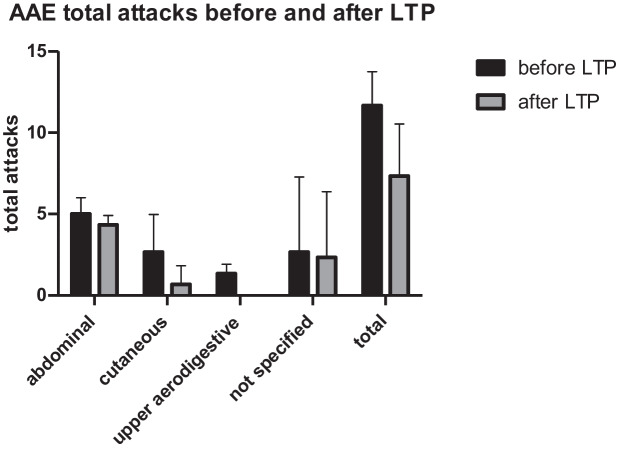
Total attacks before and after LTP with Berotralstat for AAE-C1-INH patients (*n* = 3)

**Fig. 2:**
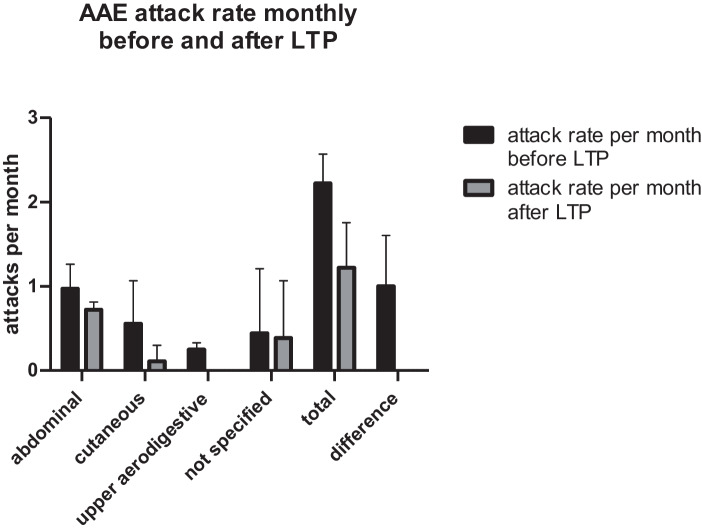
Monthly attack rate before and after LTP with Berotralstat for AAE-C1-INH patients (*n* = 3)

- The overall attack rate decreased for all the 3 AAE-C1-INH-patients from a median of 2.33 (IQR 2.08–2.42) (mean 2.22, SD ± 0.35) per month and per patient to a median of 1.0 (IQR 0.92–1.42) (mean 1.22, SD ± 0.54) per month and per patient after 6 months of LTP with Berotralstat. After LTP with Berotralstat, swellings in the upper aerodigestive tract were reduced to 0 for all the 3 patients (see Fig. 1).
- The overall attack rate for the 7 HAE type I patients (not including HAE-nC1-INH) decreased from a median of 3.33 (IQR 2.5–3.83) (mean 3.07, SD ± 0.93) per month and per patient to a median of 1.50 (IQR 0.25–2.75) (mean 1.64, SD ± 1.57) after 6 months of LTP with Berotralstat (Fig. 2).
- The two patients with HAE-nC1-INH (patients P11 and P12) went from an average of 3.83 per month to an average of 1 attack per month at 3 months. The data at 6 months for P11 could not be included because it was prospective in regard to the start of the study.
- A total of 2 patients (P6 and P8) stopped Berotralstat treatment before reaching 6 months of treatment and 1 patient (P11) was on therapy for less than 6 months at the end-timepoint of data evaluation (see description below for details):
- Patient (P6) experienced gastrointestinal side-effects including nausea, vomiting, and diarrhea after about 2 weeks of treatment with Berotralstat. She paused the LTP for 2 months and then decided to resume again. Treatment was continued for a further 3 months with repeated attacks of diarrhea and 1 reported HAE-attack per week, so she was switched to LTP with Lanadelumab.
- Patient (P8) discontinued Berotralstat after 3 months because of a lack of efficacy with no change at all in attack rates.
- Patient (P11) with HAE-nC1-INH started therapy 4 months before the timepoint of this evaluation.

### Changes in Quality of Life (Before and After LTP with Berotralstat)

**Fig. 3:**
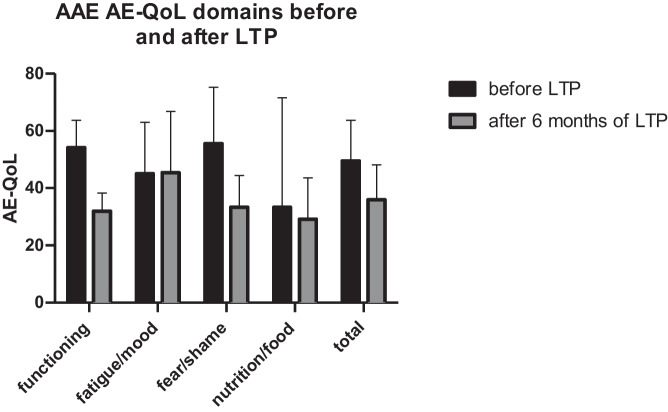
Attack rate per domain before and after LTP with Berotralstat for AAE-C1-INH patients (*n* = 3)

**Fig. 4:**
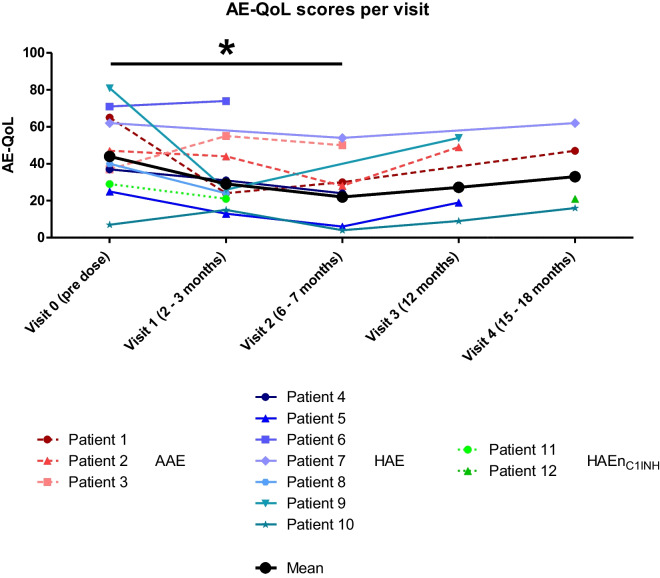
Total AE-QoL for all the patients AAE-C1-INH and HAE (*n* = 12) over the whole observation period

**Fig. 5:**
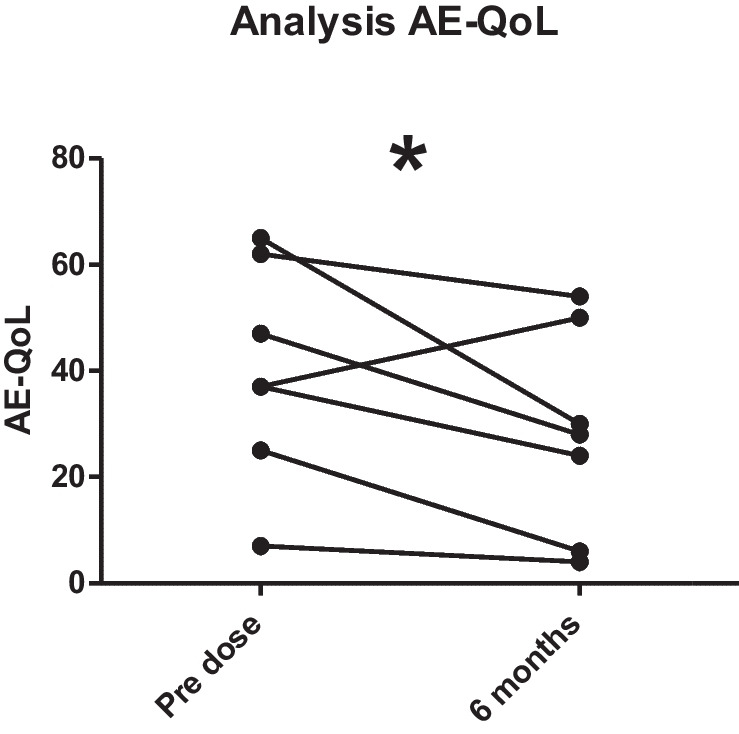
Analysis of AE-QoL scores pre-dose and after 6 months using Wilcoxon matched pairs test signed rank test (*p* < 0.0449)

- The total AE-QoL for the 3 AAE-C1-INH patients showed a mean reduction of 13.67 points (from 49.67 to 36.00) after 6 months of Berotralstat. Within the single domains, the most noticeable reductions could be found for the category ‘functioning’ with a mean reduction of 22.22 (from 54.17 to 31.94 and for ‘fear/shame’ with a mean reduction of 22.22 (from 55.55 to 33.33). A change of at least 6 is considered to be the minimal clinically important difference by the developers of AE-QoL. The scores for the domains ‘nutrition/food’ and ‘fatigue/mood’ showed a change of less than 6 points (see Fig. 3).
- For the included 7 HAE type I patients (not including HAE-nC1-INH), there was a lack of data for the single answers of the AE-QoL either before or after the start of LTP with Berotralstat; therefore, the single domains of AE-QoL could be evaluated only for 5 patients (P4, P5, P6, P7, P10).
- AE-QoL data was only available for one patient with HAE-nC1-INH at 3 months, showing a lowered AE-QoL score from 29 to 21.
- When including both HAE type I and HAE-nC1-INH patients, the data for the total AE-QoL was available for 8 of the 9 HAE patients and showed a reduction of 14.86 after 3 months (from mean of 44 at baseline (*n* = 8) to 29.14 after 3 months (*n* = 7)) and a reduction of 22.00 after 6 months of therapy (mean of 22 after 6 months (*n* = 4)) (Fig. 4). A change of at least 6 is considered to be the minimal clinically important difference by the developers of AE-QoL.
- When including AAE and all HAE patients (type I and HAE-nC1-INH) over the whole observation period, 10 out of 12 patients reached at least once an AE-QoL score of less than 40 as sign of slight impairment of quality of life.
- A Wilcoxon matched-pairs signed rank test on analysis of AE-QoL pre-treatment and after 6 months of treatment demonstrated a significant (*p* < 0.0449) improvement in scores (Fig. 5).

### Changes in Disease Control (Before and After LTP with Berotralstat)

**Fig. 6:**
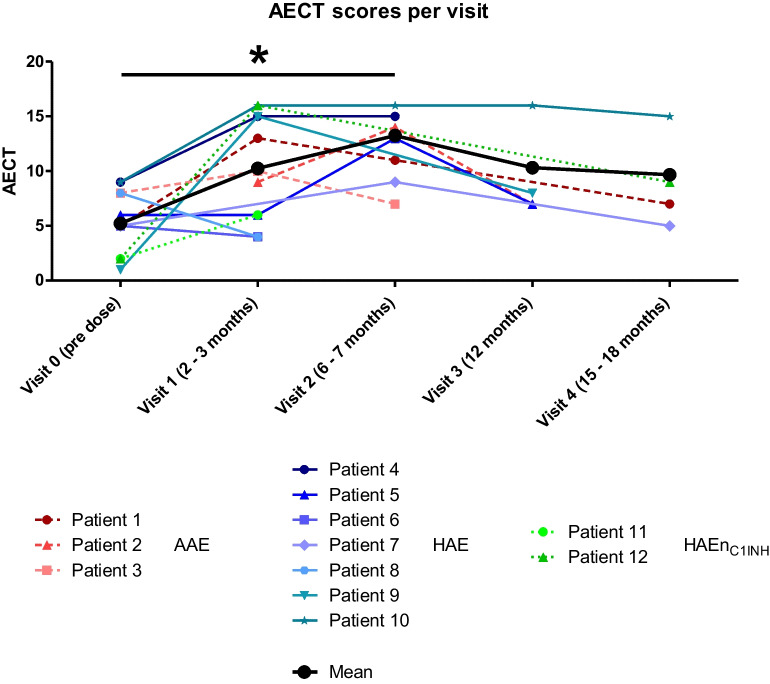
Total AECT for all the patients AAE-C1-INH and HAE (*n* = 12) over the whole observation period

**Fig. 7:**
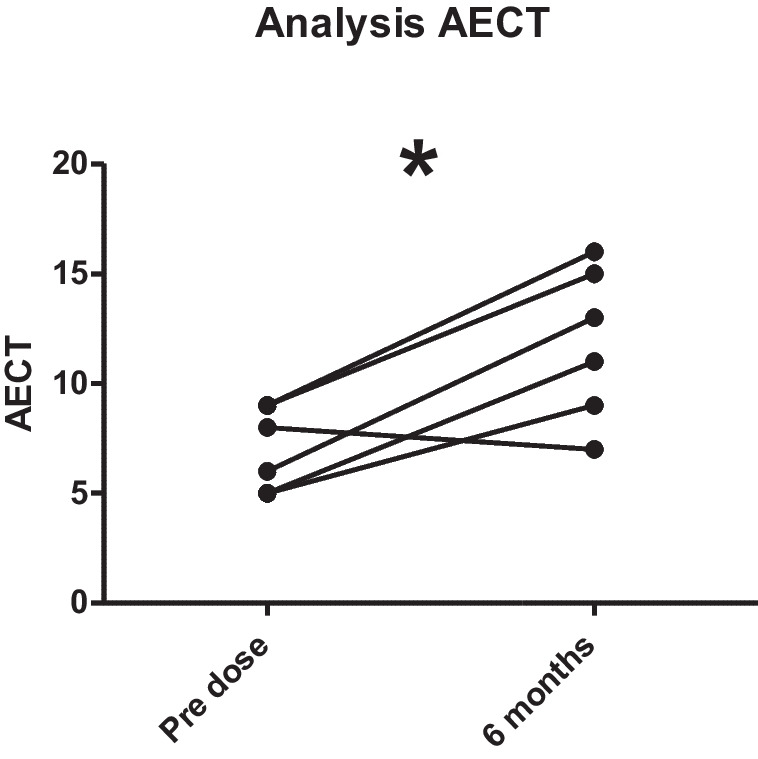
Analysis of AECT scores pre-dose and after 6 months using Wilcoxon matched pairs test signed rank test (*p* < 0.0289)

- Disease control was measured using the validated Angioedema Control Test (AECT) survey [25]. Disease control measured with AECT increased from a mean of 6.5 points to a mean of 10.7 points after 3 months and remained the same after 6 months of LTP with Berotralstat for the 3 AAE-C1-INH patients, reaching the cut-off value of 10 points (Fig. 6). One patient (P3) reached the cut-off value of 10 already after 2 months of LTP with Berotralstat, but after a total of 6 months, her status worsened and AECT decreased to 7, which lead to the decision to change the LTP. The decision to change the LTP was due to persisting angioedema attacks and concurrent problems with therapy compliance. The other two patients stayed over the cut-off value of 10.
- For the HAE type I patients (*n* = 7; not including HAE-nC1-INH), values for AECT were available for baseline and after 3 months of LTP with Berotralstat resulting in an increase of 3.86 points (from a mean of 6.14 SD ± 2.64 to a mean of 10.00 SD ± 5.39). For the timepoint after 6 months of therapy, only questionnaires of 4 patients were available, showing a mean AECT of 13.25 (SD ± 2.68).
- AECT scores were only available for HAE-nC1-INH patients at 0 and 3 months. A mean increase of 9 was noted (from 2.00 to 11.00).
- When including both AAE and all HAE patients (type I and HAE-nC1-INH) over the whole observation period, 8 out of 12 patients reached at least once an AECT score of more or equal 10. Seven patients, including 2 AAE-C1-INH patients, reached a treatment duration of 12 months with AECT available for four of them resulting in a mean of 9.50 (SD ± 4.36) (*n* = 4).
- A Wilcoxon matched-pairs signed rank test on analysis of AECT pre-treatment and after 6 months of treatment demonstrated a significant (*p* < 0.0289) improvement in scores (Fig. 7).

## Discussion

Real-world data concerning treatment with Berotralstat is limited [7, 8]. AAE-C1-INH is a rare disease and very few studies have been performed to date examining the use of LTP in patients with AAE-C1-INH. Therefore, no officially approved therapy and no guideline exists for this condition—except the treatment of the underlying disease if possible. It is rare that physicians have the opportunity to treat multiple patients of this entity, leading to a lack of real-world data. In order to learn more about this disease and how to appropriately treat it, researchers and physicians have to rely on small patient samples. While the number of patients in this study appears small, it must be considered that all analyses about patients with AAE-C1-INH are a further benefit.

An examination of contemporary literature regarding AAE-C1-INH and treatment options included case studies and small cohorts of patients. One study found a reduced overall rate of attack frequency and an improvement in AE-QoL scores in treatment of a patient with AAE-C1-INH using pd-C1-INH (Cinryze) [15]. A small cohort of 3 patients were found to have lowered attack frequency when given the kallikrein-inhibitor Lanadelumab over an 18-month time period [26]. In two of the three patients, no further attacks were noted, and dosing was advanced to once a month. The third patient experienced a 75% reduction in attacks. In a further study including 2 patients with AAE-C1-INH, a LTP treatment with Lanadelumab led to complete disease control with a maximum of 16 points in the AECT [18].

The successful on-demand administration of pd-C1-INH substitute has been described [2]. However, some patients receiving pd-C1-INH tend to become progressively non-responsive toward treatment. A tendency toward refractoriness in patients with AAE-C1-INH may be explained by the auto-cleavage from C1-Inhibitor antibodies [14, 2, 27]. This pathophysiology would support a superior effect through non-C1-INH substitutes. Rapid improvement after on-demand treatment with the B2 receptor antagonist Icatibant has also been reported in a small cohort of patients with AAE-C1-INH [16].

All current treatment options in HAE in general are applied either subcutaneously or intravenously, making Berotralstat unique. The primary recommendation for a LTP is total disease control with a normalization of patients’ lives [28]. Particularly, vulnerable patients include the elderly who live alone and are socially isolated. Obese patients with poor peripheral vascular status often have difficulties in the application of intravenous substrate C1-INH. These patients, as well as those with a needle phobia had until recently no other options in the treatment of their HAE or AAE.

In this study, patients with AAE-C1-INH were aged 49, 77, and 50 at diagnosis, with a respective reported time from onset of symptoms to diagnosis of 12, 15, and 74 months. The median age described in other studies varies between 56 [14] and 73.5 years [5], with a median time between symptom onset and diagnosis varying between 7.5 months [29] to 2 years [5]. Very little data is currently available concerning patients with either HAE-nC1-INH or AAE-C1-INH and treatment with Berotralstat.

### Changes in Attack Rate (Before and After LTP with Berotralstat)

- After 6 months of treatment, an overall attack rate decrease was seen in the AAE-C1-INH group with a median attack rate decreased from 2.33 to 1.0 per month. Furthermore, no aerodigestive attacks were described for all three AAE-C1-INH patients after 6 months of treatment. This suggests that Berotralstat works well in reducing the frequency of angioedema attacks including aerodigestive attacks in the rare subgroup of patients with AAE-C1-INH.
- In the HAE population, HAE attacks were also seen to decrease (median of 3.33 to 1.50 per month) after 6 months of treatment. Two dropouts were recorded in the first 6 months, one due to reported gastrointestinal side-effects (patient P6) and one due to a lack of reduction in recorded attacks (patient P8).

In the phase-3 Berotralstat study, gastrointestinal side-effects including abdominal pain and diarrhea were among the most common reported side-effects. The phase-3 study reported a significant attack reduction after a 6-month period from 3.00 to 1.31 per month (net reduction of 1.69) [6].

- In comparison, the median net reduction in attacks in the HAE type I population of this study was 1.8 and in the AAE-C1-INH population 1.00 attacks.
- The 2 patients with HAE-nC1-INH (patients P11 and P12) went from 2.17 attacks per month to 0 attacks after 3 months of treatment (P11) and from 5.5 attacks per month to 2.0 attacks per month and reduced attack severity reported after 6 months of treatment (P12). These results corroborate those found in one case study and one study involving 3 patients with HAE-nC1-INH.

A case study described treatment with Berotralstat in a patient with HAE-nC1-INH. Treatment was initiated due to needle phobia, and reported a reduction of an average attack rate of 2 per month to no attacks at all after 6 months of treatment. The use of questionnaires was not described [23]. The largest study to date of patients with HAE-nC1-INH angioedema (*n* = 3) treated with Berotralstat also reported a reduction in attacks and significant improvement in quality of life using the AECT and AE-QoL questionnaires [30].

### Changes in Quality of Life (Before and After LTP with Berotralstat)

- After 3 months of treatment, a mean reduction of 8.7 was noted in the AAE-C1-INH group, and after 6 months a reduction of 13.7.
- Three months of treatment revealed a mean reduction of 15.6 points in the HAE type I group, with a reduction of 24.1 (from 46.1 to 22.0) following 6 months of care.
- Data for the AE-QoL for patients with HAE-nC1-INH was limited (*n* = 1) and showed a decrease from 29 at baseline to 21 after 3 months.
- This indicates a quick improvement after 3 months of treatment with Berotralstat in the HAE and AAE-C1-INH populations, and corresponds well with results seen in other studies [31].

### Changes in Disease Control (Before and After LTP with Berotralstat)

- For the patients with AAE-C1-INH, the mean AECT score rapidly increased from 6.5 points to 10.7 points after 3 months and remained at this level until after 6 months. No other studies have reported data concerning AECT scores and treatment of patients with AAE-C1-INH with Berotralstat.
- The mean AECT score for HAE type I patients (*n* = 7) at baseline was 6.1; this increased to 10.0 after 3 months of treatment, and to 13.3 after 6 months of treatment. This confirms results reported from other studies concerning real-world data [7]. After the 6-month interval, a slight drop in scores was noticed, which may be due to a subjective decrease in satisfaction as patients strive for less to no attacks and therefore satisfaction decreases again, which is reflected in the questionnaires.
- AECT scores for HAE-nC1-INH patients (*n* = 2) at 0 and 3 months demonstrated a mean increase of 9 (from 2.0 to 11.0). This corroborates results from a case series with 3 patients with HAE-nC1-INH [30].

This study reports on real-world data concerning the treatment of patients with Berotralstat in Germany. Included in this cohort were the rare disease entities HAE-nC1-INH and AAE-C1-INH. This is the first study documenting the use of Berotralstat in patients who have AAE-C1-INH, for which no clinical data is currently available. Despite missed visits from some patients and a drop-out due to the known gastrointestinal side effects of Berotralstat, it is still apparent that a quick and sustained improvement in attacks and quality of life is achieved. The real-world data presented was collected from a heterogeneous group of patients. Two of these patients were switched to Berotralstat due to a loss of clinical effectivity from a LTP with Lanadelumab.

Over the course of the treatment with Berotralstat, patients with HAE, HAE-nC1-INH, and AAE-C1-INH had reduced angioedema attacks and an improved quality of life. The data strongly suggest that Berotralstat is able to quickly reduce angioedema attacks also in real-world application outside of controlled clinical trials. Patients had a sustained and clinically meaningful improvement in QoL surveys across multiple subpopulations.

## Conclusion

Berotralstat is the first oral treatment available for patients with hereditary angioedema. No data is currently available for patients with AAE-C1-INH and a treatment with Berotralstat. This study demonstrates a reduction of angioedema attacks and subsequent improvement in the quality of life in patients with hereditary angioedema including patients with HAE-nC1-INH and in patients with AAE-C1-INH. To help determine the efficacy of treating patients with AAE-C1-INH using Berotralstat larger comparative multi-institutional observational studies are warranted.

## Abbreviations

AAE: Acquired angioedema
C1-INH: C1-inhibitor
C1-INH-a: C1-inhibitor antigen
C1-INH-f: C1-inhibitor function
AAE-C1-INH: Acquired C1-inhibitor-deficiency
HAE: Hereditary angioedema
HAE-nC1-INH: Hereditary angioedema with normal C1-inhibitor
pdC1-INH: Plasma-derived C1-inhibitor
rhC1-INH: Recombinant human C1-inhibitor
LTP: Long-term prophylaxis
ODT: On-demand therapy
